# Characterization of Two Novel *Gammapapillomaviruses*, HPV179 and HPV184, Isolated from Common Warts of a Renal-Transplant Recipient

**DOI:** 10.1371/journal.pone.0119154

**Published:** 2015-03-06

**Authors:** Lea Hošnjak, Boštjan J. Kocjan, Branko Pirš, Katja Seme, Mario Poljak

**Affiliations:** 1 Institute of Microbiology and Immunology, Faculty of Medicine, University of Ljubljana, Ljubljana, Slovenia; 2 Private Center for Laser and Aesthetic Dermatology, Ljubljana, Slovenia; National Institute of Health - National Cancer Institute, UNITED STATES

## Abstract

*Gammapapillomavirus* (*Gamma*-PV) is a diverse and rapidly expanding PV-genus, currently consisting of 76 fully characterized human papillomavirus (HPV) types. In this study, DNA genomes of two novel HPV types, HPV179 and HPV184, obtained from two distinct facial *verrucae vulgares* specimens of a 64 year-old renal-transplant recipient, were fully cloned, sequenced and characterized. HPV179 and HPV184 genomes comprise 7,228-bp and 7,324-bp, respectively, and contain four early (E1, E2, E6 and E7) and two late genes (L1 and L2); the non-coding region is typically positioned between L1 and E6 genes. Phylogenetic analysis of the L1 nucleotide sequence placed both novel types within the *Gamma*-PV genus: HPV179 was classified as a novel member of species *Gamma*-15, additionally containing HPV135 and HPV146, while HPV184 was classified as a single member of a novel species *Gamma*-25. HPV179 and HPV184 type-specific quantitative real-time PCRs were further developed and used in combination with human *beta*-globin gene quantitative real-time PCR to determine the prevalence and viral load of the novel types in the patient’s facial warts and several follow-up skin specimens, and in a representative collection, a total of 569 samples, of HPV-associated benign and malignant neoplasms, hair follicles and anal and oral mucosa specimens obtained from immunocompetent individuals. HPV179 and HPV184 viral loads in patients’ facial warts were estimated to be 2,463 and 3,200 genome copies per single cell, respectively, suggesting their active role in the development of common warts in organ-transplant recipients. In addition, in this particular patient, both novel types had established a persistent infection of the skin for more than four years. Among immunocompetent individuals, HPV179 was further detected in low-copy numbers in a few skin specimens, indicating its cutaneous tissue tropism, while HPV184 was further detected in low-copy numbers in one mucosal and a few skin specimens, suggesting its dual tissue tropism.

## Introduction

Human papillomaviruses (HPVs) are a large and heterogeneous family of small non-enveloped viruses, with circular double stranded DNA genome, and are etiologically linked with various benign and malignant neoplasms of the skin and mucosa [1]. Based on the nucleotide similarity in the L1 open reading frame (ORF), HPVs are classified into genera, species and types, and HPV types are those that are typically associated with specific disease(s) [2]. A novel HPV type is recognized as such when its complete genome has been cloned into plasmid vector(s) and the L1 ORF sequence differs by more than 10% from the closest known HPV [1]. The International HPV Reference Center at the Karolinska Institute, Stockholm, Sweden, confirms DNA sequences of novel HPV types, assigns HPV type numbers, and deposits and maintains the reference clones. As of 1 November 2014, 195 HPV types had been completely sequenced and officially recognized (http://www.hpvcenter.se/html/refclones.html). All HPV types are presented within five papillomavirus (PV) genera, including *Alphapapillomavirus (Alpha-*PV), predominantly found in the anogenital region, and *Betapapillomavirus (Beta-*PV), *Gammapapillomavirus (Gamma-*PV), *Mupapillomavirus* (*Mu-*PV), and *Nupapillomavirus* (*Nu-*PV), typically detected in skin and hair follicle specimens [3].

*Gamma-*PV is a diverse and rapidly expanding PV-genus, currently consisting of 76 officially recognized HPV types (http://www.hpvcenter.se/html/refclones.html). In addition, as shown by recent phylogenetic comparisons of partial HPV L1 gene sequences available in the GenBank database, at least 159 putative novel *Gamma-*PV types exist *in vivo* [4]. The discovery boom of novel *Gamma-*PVs in the last few years is a consequence of the application of sequence independent amplification techniques [5], novel PCR primers specifically designed to detect a broader range of *Gamma-*PVs [6, 7] and/or high-throughput sequencing methods [8–13]. *Gamma-*PV types seem to be ubiquitous in healthy skin worldwide [9, 10, 13, 14]. Additionally, growing evidence indicates that these viruses can be present not only in skin specimens but also at various mucosal sites, including the oral and nasal cavity, penis and anal canal [15–17].

Cutaneous warts are the most frequent benign lesion of the skin caused by HPV. In immunocompetent subjects, these lesions are predominately caused by HPV types from *Alpha*-PV species *Alpha*-2 (HPV3, 10, 28, 29, 77, 78, 94, 125, and 160) and *Alpha*-4 (HPV2, 27 and 57), and less frequently also by HPV types from *Gamma-*PV (HPV4), *Mu-*PV (HPV1 and 63) and *Nu-*PV (HPV41) genera [2, 18–20]. In immunosuppressed patients, it seems that cutaneous warts are etiologically linked with some additional *Alpha*-PVs (e.g. HPV117 and HPVXS2) and a more diverse range of *Gamma-*PVs, including HPV60, 95, 126, 138 and 149 [2, 18–24].

In the present study, we report the genomic characterization and phylogenetic evaluation of two novel *Gamma-*PV types, HPV179 and HPV184, originally detected in common warts of a renal-transplant recipient that had previously tested negative for the major cutaneous wart causing HPVs. In addition, a representative collection of HPV-associated benign and malignant neoplasms, hair follicles and anal and oral mucosa specimens was tested, using HPV179 and HPV184 quantitative type-specific real-time PCRs, in order to determine the tissue tropism and potential clinical relevance of HPV179 and HPV184.

## Materials and Methods

### Patient and skin wart specimens

In 2010, four years after the renal transplantation, two common warts developed on the cheeks (one on each side) of a 64 year-old male patient. Both warts were removed six months after their occurrence by excisional biopsy; one part of each specimen was stored in liquid nitrogen for DNA extraction and molecular analysis, while the other part was fixed with formalin and embedded into paraffin for histopathological examination.

In 2014, the patient returned due to the development of a common wart on the neck, which was removed three months after its occurrence by excisional biopsy and processed as described above. The patient reported no recurrence of facial common warts following their removal in 2010. In addition, a pooled hair follicle sample from both eyebrows, a swab of the forehead, swabs of the left and right cheek, and the swab sample surrounding the removed tissue on the neck, were obtained, using 4NG FLOQSwabs (Copan, Brescia, Italy), certified as DNase, RNase-Free and Human DNA-Free (Table 1).

**Table 1:** Characteristics of samples obtained from an immunosuppressed patient.

Tissue type	Location	Histology (sample type)	Sample origin	HPV179 viral load (viral copies/cell)^a^	HPV184 viral load (viral copies/cell)^a^	Presence of other HPVs
Cutaneous	Right cheek	Common wart (fresh tissue)	original sample	0.30	3,200	8, 9, 23, 49, 150 (subtype), 174, 179
	Left cheek	Common wart (fresh tissue)	original sample	2,463	0.40	8, 9, 12, 14, 15, 20, 23, 24, 49, 75, 80, 93, 150 (subtype), 174, 184
	Right cheek	Healthy skin (swab)	follow-up	HPV179-positive	HPV184-positive	9, 14, 15, 20, 23, 24, 49, 75, 80, 115
	Left cheek	Healthy skin (swab)	follow-up	HPV179-positive	HPV184-positive	9, 12, 14, 15, 23, 24, 36, 49, 115
	Neck	Common wart (fresh tissue)	follow-up	0.01	0.20	9, 15, 23, 24
	Neck	Skin adjecent to the wart (swab)	follow-up	HPV179-positive	HPV184-positive	9, 14, 15, 20, 23, 24, 49, 115
	Forehead	Healthy skin (swab)	follow-up	HPV179-positive	HPV184-positive	9, 14, 15, 23, 80
	Eyebrow hairs	Eyebrows (hair follicles)	follow-up	0.06	1.59	9, 20, 23, 24, 49

^a^ Swab samples were only determined as HPV179/-184 positive.

### DNA extraction and amplification of partial viral sequences

Total DNA was extracted from fresh wart tissue specimens using a QIAamp DNA Mini Kit (Qiagen, Hilden, Germany), following the manufacturer’s instructions, and quantified by NanoDrop ND-2000c spectrophotometer (NanoDrop Technologies, Oxfordshire, UK). Total DNA was extracted from hair follicle specimens using a High Pure PCR Template Preparation Kit (Roche Diagnostics, Mannheim, Germany), as described previously [25]. Total DNA was extracted from skin swab specimens using an EZ1 Advanced Instrument (Qiagen) and EZ1 DNA Investigator Kit (Qiagen), following the slightly modified protocol: Pre-treatment for Forensic Surface and Contact Swabs. Briefly, swabs were first incubated for 30 min at 56°C with 400 μl of water diluted by Buffer G2 (ratio 1:1) and 10 μl of proteinase K. After the addition of 1 μl of carrier RNA (1 μg/μl), extraction of DNA was carried out following the EZ1 robot Tip Dance protocol. Final elution volumes of the bound DNA were 100 μl for the tissue samples and 50 μl for hair follicles and skin swab samples. The quality and concentration of extracted DNA was determined by quantitative RT-PCR amplification of a 150-bp fragment of human *beta*-globin gene using beta-403f/beta-532r primers, as described previously [26].

Initial testing of both common warts was performed using HVP2/B5 and »low-risk« *Alpha*-PV PCR protocols [18, 27], together enabling the detection of 26 HPV types from *Alpha-*PV, *Mu-*PV and *Nu-*PV genera that are typically associated with various mucosal and cutaneous warts. For the detection of *Gamma*-PV types, PCR amplification was additionally performed using a recently developed E1Gamma-F and E1Gamma-R primer set, enabling amplification of an approximately 500-bp fragment of the E1 gene of a wide spectrum of *Gamma-*PV types [6]. Briefly, a PCR reaction was performed on a Veriti Thermal Cycler (Applied Biosystems, Foster City, USA) using a HotStarTaq Plus DNA Polymerase kit (Qiagen). The reaction mixture contained 100 ng of extracted tissue DNA, 2.5 μl of CoralLoad PCR Buffer with 15 mM MgCl_2_, 200 μM of dNTPs, 0.625 U of HotStarTaq Plus DNA polymerase, 0.45 μM of each primer and water up to 25 μl. The cycling conditions used were 95°C for 5 min, followed by 45 cycles of 94°C for 30 sec, 52°C for 30 sec and 72°C for 1 min, followed by final extension at 72°C for 10 min and cooling the reaction mixture to 8°C. The obtained PCR products were gel-purified and further processed for sequence analysis and HPV type determination, as described previously [25].

### Amplification, cloning and sequencing of HPV179 and HPV184 genomes

Complete genomes of HPV179 and HPV184 were initially pre-amplified using rolling circle amplification (RCA) [5] and then PCR-amplified using two overlapping primer sets. Specifically, HPV179 was amplified using the HPV broad-range primers: *Gamma-*PV-E1F and FAP64 [6, 28], and FAP6085F and *Gamma-*PV-E1R [6, 29], resulting in 3,819-bp and 4,282-bp fragments, respectively. HPV184 was amplified using HPV184 type-specific primers: SIBX17-LNG-F3 (5'-TAGATGTGTTTGCAGAAGAAAGC-3', nt 2,257–2,279) and SIBX17-LNG-R3 (5'-CCATAAACTGCCAACAAGGAAA-3', nt 2,151–2,130), and broad-range primers *Gamma-*PV-E1F and *Gamma-*PV-E1R, resulting in 7,219-bp and 505-bp fragments, respectively. All PCR reactions were performed on a Veriti Thermal Cycler (Applied Biosystems) using a Platinum *Taq* DNA Polymerase High Fidelity Kit (Invitrogen, Carlsbad, USA). The reaction mixture contained 10 μl of water diluted RCA product (1:100), 5 μl of 10X High Fidelity PCR Buffer, 2 μl of 50 mM MgSO_4_, 200 μM of dNTPs, 1 U of Platinum Taq DNA Polymerase High Fidelity, 0.2 μM of each primer, and water up to 50 μl. The cycling conditions used for all long range PCRs were 94°C for 2 min, followed by 45 cycles of 94°C for 30 s, 59°C for 30 s and 68°C for 1 minute per kb of PCR product, followed by a final extension at 68°C for 7 min and cooling the reaction mixture to 8°C.

The obtained PCR products were analyzed on a 0.8% agarose gel using E-Gel 96 High Range DNA Marker (Invitrogen) and purified with a QIAquick PCR Purification Kit (Qiagen). Plasmid clones, containing overlapping viral genome fragments, were prepared using a TOPO XL PCR Cloning Kit (Invitrogen) and transformed into enclosed One Shot TOP10 Chemically Competent *E*. *coli* strain, following the manufacturer’s instructions. Plasmid DNA of each clone was extracted from 4 ml of LB kanamycin medium containing overnight grown bacterial culture using a QIAprep Spin Miniprep Kit (Qiagen), following the manufacturer’s instructions. The extracted DNA was quantified and sequenced using the both strand primer-walking strategy at Microsynth AG (Balgach, Switzerland).

### Genome characterization and phylogenetic analysis

The complete viral genomes of HPV179 and HPV184 were assembled using the Vector NTI Advance 11 program package (Invitrogen). HPV specific ORFs were determined using the ORF Finder Tool (http://www.ncbi.nlm.nih.gov/gorf/gorf.html). Published literature and several Web-based free of charge applications were additionally used to characterize in detail the HPV179 and HPV184 ORFs and proteins, and non-coding long control region (LCR), including Poly(A) Signal Miner [30], SIGSCAN software V4.05 [31], 2ZIP—Server [32], GPMiner (http://gpminer.mbc.nctu.edu.tw/index.php), and programs at Papillomavirus Episteme database [33].

The phylogenetic analysis was based on the entire L1 gene sequences of HPV179 and HPV184 and all currently completely sequenced PVs from the *Gamma-*PV genus and closely related animal PV genera, including *Dyoxipapillomavirus*, *Phipapillomavirus*, *Pipapillomavirus*, *Taupapillomavirus*, *Omikronpapillomavirus*, *Upsilonpapillomavirus* and *Xipapillomavirus*, and rooted with *Beta-*PVs, HPV5 and -8. All eligible PV sequences were obtained through the Papillomavirus Episteme database [33] and aligned using MAFFT v6.846 software [34]. The best suitable evolutionary model, General Time Reversible (GTR) with discrete Gamma distribution (+G) with 5 rate categories and by assuming that a certain fraction of sites are evolutionary invariable (+I), was selected using a MEGA5 software package [36]. The same software was additionally used to construct the subsequent maximum likelihood tree, employing 1,000 bootstrap values. Additionally, maximum likelihood tree was constructed using RAxML HPC2 v7.6.3 [35], employing 1,000 bootstrap values, using GTRCAT model for bootstrapping phase. The phylogenetic tree was visualized and acquired with the MEGA5 software package [36].

In order to compare the genomes of HPV179 and HPV184 with the genomes of all phylogenetically related HPV types, additional pairwise nucleotide and amino acid sequence alignments were carried out and percentage similarity was calculated for all viral genes shared between novel HPV types and each of the related types, using the EMBOSS Water Pairwise Sequence Alignment tool (http://www.ebi.ac.uk/Tools/psa/emboss_water/). The PASC software [37], integrated into the NCBI database, was additionally used to determine HPV179 and HPV184 whole genome pairwise identity with closely related HPV types.

### HPV179 and HPV184 type-specific quantitative real-time PCR assays

HPV179 and HPV184 type-specific primers and dually labeled Taqman probes were designed within partial E1 gene sequences (ENA acc. nos. HG530536 and HG530537), using on-line primer/probe design software (http://eu.idtdna.com/PrimerQuest/Home/Index), resulting in PCR products of 128-bp and 150-bp in length, respectively. Primers SIBX16-RTPCR-F (5'-TCAATGATGAATCGCCATAGTC-3', nt 2,047–2,068) and SIBX16-RTPCR-R (5'-CCGTCTAACGCAGCTCTCA-3', nt 2,174–2,156) together with 5’-FAM/ZEN-labeled SIBX16-RTPCR-P probe (5’-TTTTGGTTAATGCCACTACAGGATGGT-3', nt 2,071–2,097) were used to detect HPV179. Primers SIBX17-RTPCR-F (5'-TTTCCTTGTTGGCAGTTTATGG-3', nt 2,130–2,151) and SIBX17-RTPCR-R (5'-GCTTTCTTCTGCAAACACATCTA-3', nt 2,279–2,257) together with 5’-HEX/ZEN-labeled SIBX17-RTPCR-P probe (5’-CAAAGCACCTATTCAGATGAAATTACCACC-3’, nt 2,207–2,236) were used to detect HPV184. The specificity of the selected primers and probes was verified *in-silico* using NCBI Blast and MFEprimer-2.0 (http://biocompute.bmi.ac.cn/CZlab/MFEprimer-2.0/index.cgi) web-based services. Using these algorithms, and by sequencing all HPV179 and HPV184 RT-PCR amplicons, obtained later in the study, it was confirmed that neither non-targeted PV sequences or human DNA can be amplified/detected with these primers/probes.

The detection of HPV179 and HPV184 was performed in two separated type-specific RT-PCR assays using a LightCycler 480 Probes Master Kit and LightCycler 480 II RT-PCR Instrument (Roche). Briefly, the final, thoroughly optimized RT-PCR assay was performed in a 96-well plate, with each reaction well containing 5 μl of template DNA (tissue samples 50–100 ng), 10 μl of 2X LightCycler 480 Probes Master, 0.5 μM of each primer, 0.2 μM of each probe, and water up to 20 μl. The cycling conditions were the same for both novel HPV types and were as follows: initial DNA denaturation at 95°C for 10 min, followed by 40 amplification cycles of 95°C for 10 s, 60°C for 30 s and 72°C for 1 s. A final step consisted of cooling at 4.4°C/s to 40°C with a 30 s hold. The temperature transition rate was set to 4.4°C/s for all RT-PCR steps. Real-time monitoring of the fluorescent signal was performed on the 530 (HPV179) and 640 (HPV184) nm channels.

Testing triplicates of 10-fold diluted plasmids, spanning from 1 × 10^9^ to 1 × 10^0^ DNA copies per reaction in a background of 100 ng of commercially available human DNA (Human Genomic DNA; Promega, Madison, WI), showed that both RT-PCR assays had a sensitivity of at least 10 viral genome equivalents. The dynamic range of the HPV179 and HPV184 RT-PCR assays was eight orders of magnitude, enabling reliable discrimination of 10–10^9^ viral genome equivalents per reaction. The calculated correlation coefficients (R^2^) of HPV179 and HPV184 RT-PCR assays standard curves were 0.999 and 0.988, respectively. HPV179 and HPV184 RT-PCR amplification efficiencies (E) were satisfactory and were estimated to 93.3% and 90.0%, respectively.

### Clinical samples and HPV179/HPV184 prevalence and viral load

HPV179 and HPV184 type-specific quantitative RT-PCR assays were used in combination with human *beta*-globin gene quantitative RT-PCR [38] to determine the prevalence and viral load of both novel HPV types in the original wart specimens and 569 other clinical specimens, including tissue samples of histologically confirmed common warts (94 samples), genital warts (31 samples), laryngeal papillomas (31 samples), conjunctival papillomas (31 samples), squamous cell carcinoma (SSC) of the cervix (31 samples), SCC of the oral cavity/oropharynx (50 samples), SCC of the skin (50 samples), basal cell carcinoma (BSC) of the skin (51 samples), hair follicles (100 samples), swabs of the oral cavity (50 samples) and swabs of the anal canal (50 samples). Each clinical sample was obtained from a different individual and total DNA was extracted using various, specimen-specific protocols, as described previously [25, 39, 40]. The internal control amplification with beta-403f/beta-532r primers showed that the amplifiable and good quality DNA was recovered from all 569 clinical specimens. The number of human cells for the HPV179 or HPV184 positive samples was determined based on the human *beta*-globin concentration and used to determine the ratio between the number of viral genomes and human cells. In all calculations we assumed that one cell contains 6.6 pg of DNA [41].

The presence of additional HPV types in HPV179 or HPV184 positive samples was examined using several in-house and commercial HPV broad-range PCR protocols. For all PCR experiments, 5 μl of template DNA (tissue samples 50–100 ng) was used per 25 μl reaction. Amplification products (10 μl) of each PCR assay were analyzed by electrophoresis on 2% agarose gels dyed with fluorescent SYBR Safe DNA gel stain (Invitrogen). HPV types were determined by direct sequencing of PCR products and Blast analysis [25], unless indicated otherwise. Briefly, for the detection of *Beta-*PV types, a reverse line-blot hybridization-based RHA Kit Skin (beta) HPV test (Diassay BV, Rijswijk, The Netherlands) was used, able to detect 25 different HPV types, including HPV5, -8, -9, -12, -14, -15, -17, -19, -20, -21, -22, -23, -24, -25, -36, -37, -38, -47, -49, -75, -76, -80, -92, -93 and -96. The assay was performed as described previously [27]. The presence of the most common mucosal and cutaneous wart-associated HPV types from *Alpha*-PV (HPV2, -3, -6, -7, -10, -11, -13, -27, -28, -29, -32, -40, -42, -43, -44, -57, -74, -77, -78, -91, -94, -117 and -125), *Mu-*PV (HPV1 and -63) and *Nu-*PV (HPV41) genera was investigated using two previously published PCR protocols, employing HVP2/B5 primers [18] and a »low-risk« *Alpha*-PV primer set [27]. For the detection of *Gamma*-PV types, a recently described broad-range *Gamma-*PV-E1F/*Gamma-*PV-E1R primer set [6] was used in the PCR protocol detailed above. To detect a variety of HPV types from different PV-genera, the CPI/CPIIg primer set was additionally used, as described previously [25].

To control for possible amplicon carryover contamination, one water blank was used per 10 clinical samples in all (RT-) PCR runs.

### Ethics Statement

Samples from the 64-year-old renal-transplant patient were obtained as a part of a routine diagnostic procedure which initially included clinical and histopathological examination of patient’s skin neoplasms. Since histopathological examination did not provide etiology of neoplasms, further molecular virological testing had been requested by the responsible clinician. The patient was informed about all requested diagnostic procedures and he gave written informed consents for sampling, all diagnostic procedures (including histopathological examination and molecular virological testing) and publication of his case.

In addition to the renal-transplant patient’s clinical samples, 569 additional clinical samples were used in the present study in order to determine the prevalence and tissue tropism of both novel HPV types. Anal swabs, samples of the cervical cancer, hair follicles, genital warts and common warts were obtained during our past or ongoing studies [25, 39, 42–45], in compliance with the Helsinki Declaration, approved by the Ethics Committee of the Ministry of Health of Republic of Slovenia (consent references 131/06/07, 45/04/07, 34/11/06, 83/11/09, 174/05/09, 97/11/09, 100/12/09, and 63/10/13). Written informed consent was obtained from all patients. None of the patients was sampled solely for the purpose of the present study. Additionally, samples of conjunctival and laryngeal papillomas, SCC of the oral cavity/oropharynx, and SCC and BCC of the skin were obtained from the archival tissue collection of the Institute of Pathology, Faculty of Medicine, University of Ljubljana. According to the national legislation of the Republic of Slovenia, no patient’s informed consent is needed when archival clinical samples are used for research purposes. Institutional Review Boards of the Institute of Pathology and the Institute of Microbiology and Immunology, Faculty of Medicine, University of Ljubljana, specifically approved the use of all samples in the present study. Since all archival samples were coded, patient’s identity was unknown. Only patient’s gender, age and immune status were available to researchers.

## Results and Discussion

HPV179 and HPV184 were originally detected in two distinct common wart tissue specimens, histologically classified as *verruca vulgaris*, that developed on the left and right cheeks of a 64 year-old male patient four years following the renal transplantation. Initial screening of both skin lesions using two PCR protocols [18, 27], together enabling the detection of 26 different HPV types from *Alpha-*PV, *Mu-*PV and *Nu-*PV genera that are typically associated with various mucosal and cutaneous warts, was negative. Partial viral nucleotide sequences, suggesting the presence of novel HPV types in high-copy numbers, were obtained using E1Gamma-F/E1Gamma-R primers, targeting a wide spectrum of officially recognized and putatively novel *Gamma-*PV types [6]. Specifically, a partial 461-bp E1 sequence of HPV179 (ENA acc. no. HG530536) was obtained from the lesion located on the left cheek, while a partial 467-bp E1 sequence of HPV184 (HG530537) was obtained from the lesion located on the right cheek. The complete genomes of HPV179 and HPV184 were generated using an overlapping long-range PCR approach and primer walking sequencing strategy of the cloned viral genome fragments. Two reference plasmid clones, covering the full genome of HPV179 (HG421739), were deposited in the Human Papillomavirus Reference Center at the Karolinska Institute in Sweden in July 2013, where their sequences were resequenced and the type officially named in December 2013 (http://www.hpvcenter.se/html/refclones.html). The two reference plasmid clones, covering the full genome of HPV184 (HG530535), were deposited at the same institution in September 2013 where their sequences were verified by sequencing and the type officially named in February 2014 (http://www.hpvcenter.se/html/refclones.html).

**Fig 1:**
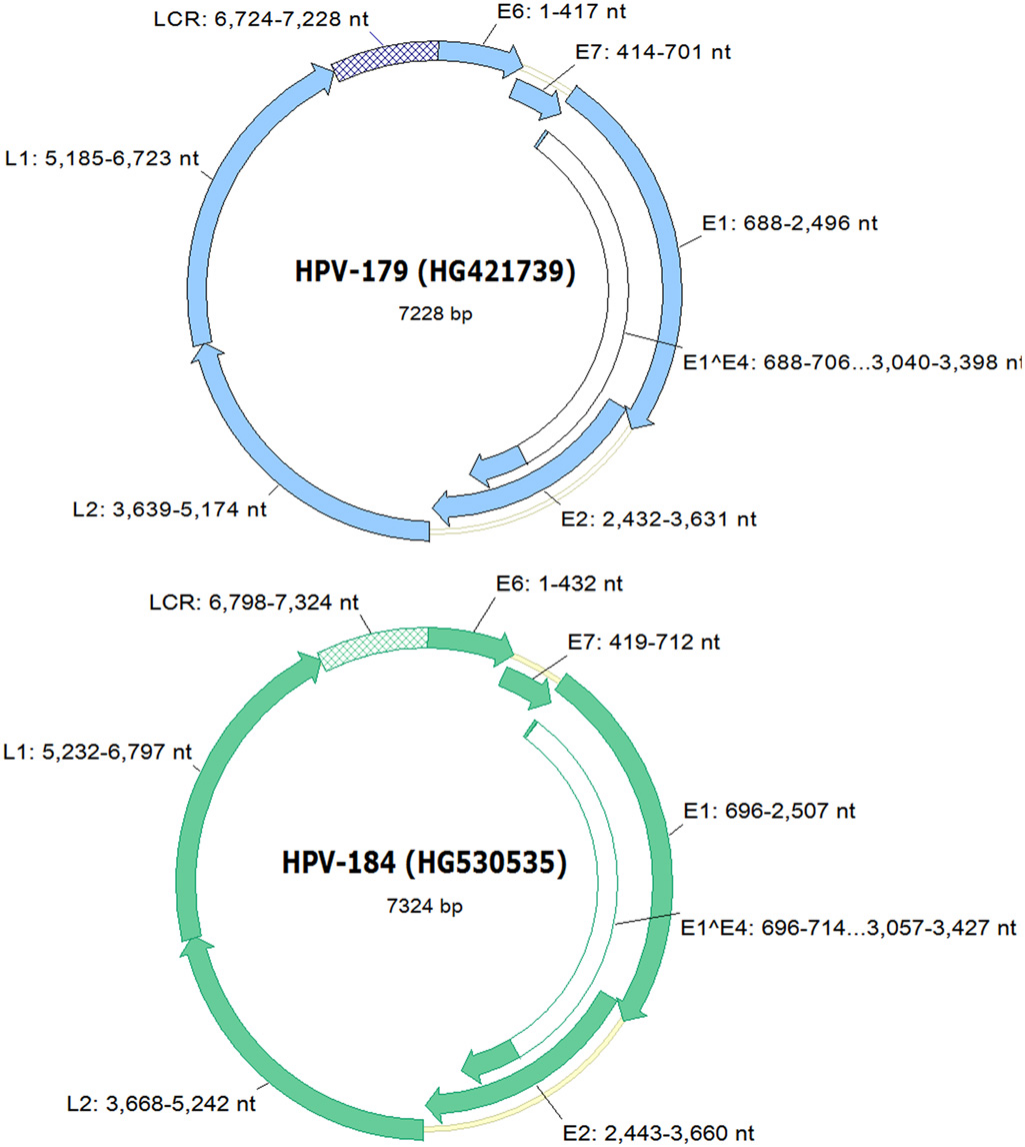
Genomic organization of HPV179 and HPV184. Genomic positions of viral genes (E6, E7, E1, E2, E4, L1 and L2) and of the non-coding region (LCR) are indicated next to the respective genomic regions.

The HPV179 genome is 7,228-bp in length and has a GC content of 36.8%, while the HPV184 genome is a few base pairs longer (7,324-bp), with a GC content of 36.9%. Both novel HPV types exhibit genome organization typical of *Gamma-*PVs and encode five early (E1, E2, E4, E6 and E7) and two late ORFs (L1 and L2) (Fig. 1), but no E5 ORF [46]. The LCRs of HPV179 and HPV184 are typically located between L1 and E6 ORFs and contain several consensus palindromic E2-binding sites (ACC-N_6_-GGT), a putative TATA box (TATAAA) of the E6 promotor, putative polyadenilation sites (AATAAA and ATTAAA) for late gene transcripts, several E1 binding sites (AACAAT), probably representing the origin of replication [20, 47], and binding sites for the transcriptional regulatory factors, including AP-1, NF-1 and C/EBPalpha [48]. In both novel HPVs, two conserved zinc-binding domains (CxxC(x)_29_CxxC), separated by 36 amino acids, were identified in the putative E6 protein and one was identified in E7. As expected, no type 1 PDZ-binding motif (S/TXV), a typical feature of the high-risk *Alpha*-HPV types [49], was identified at the extreme C-terminus of either E6 protein. Similar to the majority of *Gamma-*PV types, HPV179 and HPV184 contain no conserved cell retinoblastoma protein binding domain (LxCxE) within their putative E7 proteins. The E1 ORF encodes the largest protein of HPV179 (602 amino acid residues), as well as of HPV184 (603 amino acid residues), which contains the conserved ATP-binding site (GPPDTGKS) for the ATP-dependent helicase in its carboxyterminal part [50]. No conserved leucine-zipper domain (L-X_6_-L-X_6_-L-X_6_-L), necessary for E2 dimerization, was observed in the putative E2 proteins of novel HPV types. The putative E4 ORFs of HPV179 and HPV184 contain a start codon and completely overlap the E2 ORF. However, since we identified the characteristic donor (AAG/GUASNR) and acceptor (GUYACYAG/YU) RNA splicing sites [51], it is possible that the E4 protein of each type is translated from a spliced mRNA consisting of the first few codons of the E1 ORF joined to the E4 ORF (Fig. 1). The resulting putative E1^∧^E4 fusion proteins contain a typically high content of proline, which represents 13.6% and 14.0% of HPV179 and HPV184 E1^∧^E4 amino acid sequences, respectively.

**Fig 2:**
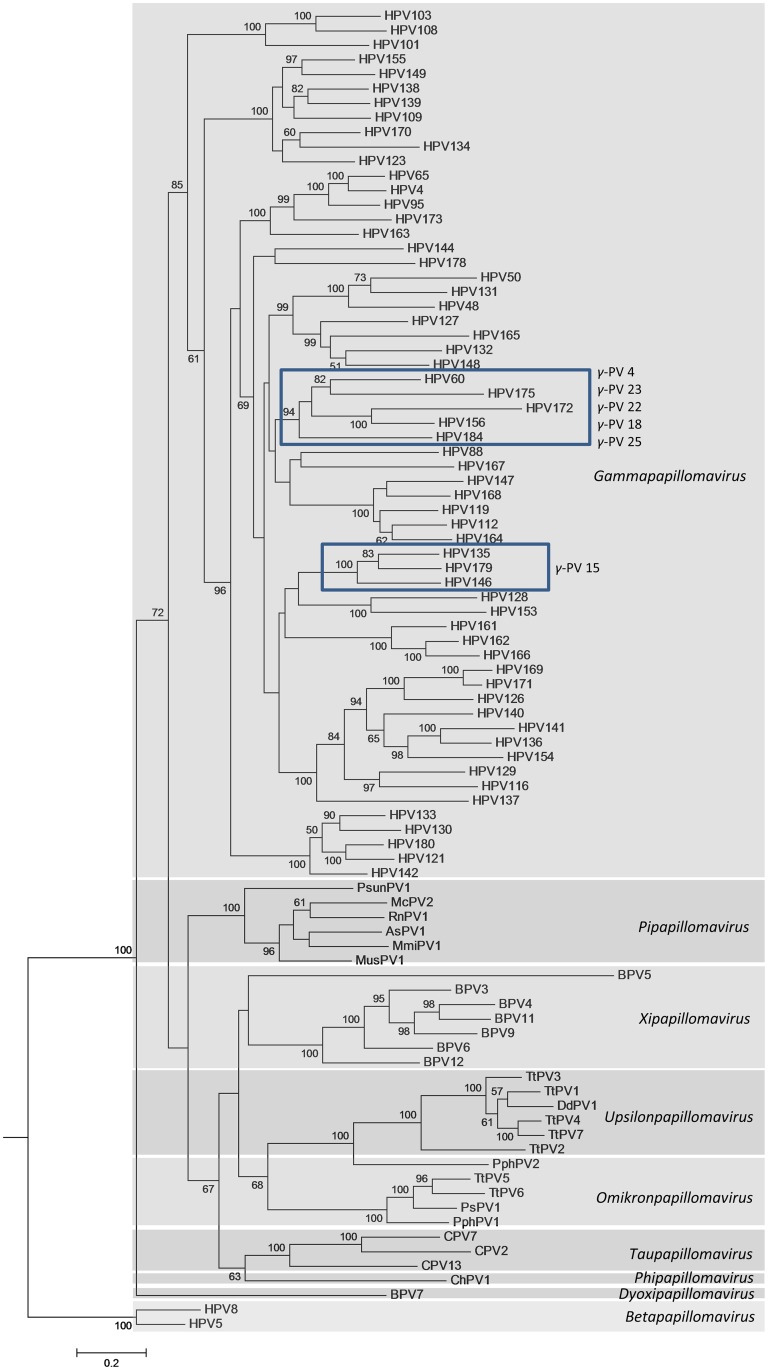
Phylogenetic position of HPV179 and HPV184. A maximum likelihood algorithm tree inferred from the L1 nucleotide sequences of HPV179 and HPV184, sixty other currently known *Gamma*-PV sequences and related animal PVs. The numbers at the internal nodes represent bootstrap support values, determined for 1000 iterations by the maximum likelihood method. Accession numbers of complete genome sequences of HPV179 and HPV184 in ENA, GenBank and DDBJ databases are presented in parentheses.

Topologies and bootstrap values of maximum likelihood phylogenetic trees, obtained with 2 distinct evolutionary models (GTRCAT and GTR+G+I), were independent of the construction method used. Therefore, only the phylogenetic tree obtained with RAxML HPC2 v7.6.3 and GTRCAT model is shown in Fig. 2. A maximum likelihood algorithm tree, inferred from L1 nucleotide sequences of HPV179 and HPV184, all currently available nucleotide sequences of officially recognized *Gamma-*PVs, and related animal PVs, confirmed that both novel HPV types belong to the genus *Gamma-*PV. As shown in Fig. 2, HPV179 is most closely related to HPV135 from species *Gamma*-15, while HPV184 probably shares a common ancestor with several HPV types, including HPV60 from species *Gamma*-4, HPV156 (*Gamma*-18), HPV172 (*Gamma*-22) and HPV175 (*Gamma*-23). Based on the established PV classification criteria [52], the HPV Reference Center identified HPV179 as a novel member of species *Gamma*-15, additionally containing HPV135 and HPV146, and HPV184 as a single member of a novel species *Gamma*-*2*5. The results of additional pairwise nucleotide and amino acid sequence comparisons of individual ORFs of HPV179 and HPV184 to the most closely related HPV types are summarized in Table 2. As shown in Table 2, HPV179 is undoubtedly the closest relative of HPV135 (PASC global alignment score: 73.99%); these two HPV types revealed the highest similarity in all of the compared ORFs and viral proteins. In contrast, in the nucleotide and amino acid sequences of HPV184 E1, E2 and L1 ORFs showed the highest similarity to the corresponding ORFs of HPV60. In contrast, the E7 ORF of HPV184 was more similar to E7 of HPV156 in both nucleotide and amino acid sequences. Interestingly, the nucleotide sequence of L2 ORF showed the highest similarity to L2 of HPV156, while its amino acid sequence was more similar to the L2 protein of HPV60. Similarly, the nucleotide sequence of E4 ORF showed the highest similarity to E4 of HPV60, while its amino acid sequence was more similar to the E4 protein of HPV175. The nucleotide sequence of E6 ORF was most similar to E6 of HPV60, while its amino acid sequence was most similar to the E6 protein of HPV172. Taken together, these findings could either reflect a recombination event or a different evolutionary divergence of HPV184, HPV60, HPV156, HPV172 and HPV175 E6 and E7 vs. E4 vs. E1, E1, L1, and L2 genes from their most recent common ancestor, similarly as recently suggested for HPV125 [38]. Nevertheless, PASC global alignment analysis, searching for the best matches within the PV family, revealed that HPV184 shares the highest average similarity (65.47%) with the whole genome of HPV60.

**Table 2:** Percentage similarity between individual genes (E6, E7, E1, E2, E4, L1, L2) of HPV179 and HPV184 and phylogenetically related HPV types.

HPV179	HPV135	HPV146	HPV184	HPV60	HPV156	HPV172	HPV175
**E6**			**E6**				
nt	76.0	71.8	nt	59.2	57.7	56.9	57.5
aa	86.9	79.4	aa	65.4	62.3	67.8	61.8
**E7**			**E7**				
nt	77.0	65.0	nt	61.0	71.3	59.0	65.8
aa	80.9	66.3	aa	78.1	79.2	70.1	78.6
**E1**			**E1**				
nt	80.9	74.5	nt	72.3	65.9	65.5	65.2
aa	90.9	85.2	aa	82.7	75.4	73.7	74.9
**E2**			**E2**				
nt	77.9	73.2	nt	62.9	60.1	60.9	61.1
aa	85.1	79.8	aa	69.5	67.6	62.2	65.2
**E4**			**E4**				
nt	75.4	70.1	nt	61.0	53.6	51.6	59.6
aa	68.7	66.2	aa	54.8	53.3	47.8	59.7
**L1**			**L1**				
nt	76.9	74.1	nt	68.0	67.2	62.4	61.7
aa	87.8	86.7	aa	81.6	79.5	71.5	76.1
**L2**			**L2**				
nt	67.6	62.7	nt	54.8	55.0	53.2	53.9
aa	78.3	72.6	aa	66.3	60.1	56.3	60.9

Abbreviations/legend: nt—nucleotide. aa—amino acid.

A recent extensive report on tissue tropism of *Gamma-*PVs indicates that the majority of these types can be found in a broader variety of anatomical locations than previously appreciated, including various mucosal, cutaneous and mucocutaneous sites. Various anatomical sites are found for members of the same *Gamma-*PV species or even type, supporting the idea that *Gamma-*PVs are in fact generalists, with various forms of tissue tropism [20]. The closest relative of HPV179, HPV135, was originally detected in a skin swab specimen of a 40-year-old renal transplant recipient [53] and later also in the oral cavity [15], nasal cavity [16] and anogenital region [17] of healthy subjects. HPV146 was initially detected in an oral rinse sample and later in healthy skin [14] and the nasal cavity [16]. The closest relative of HPV184, HPV60, was initially detected in a plantar wart and was classified as its causative agent [54]. HPV175 was identified with a metagenomic approach as a single HPV type in a swab sample of a genital wart that had previously tested HPV negative using various broad-range PCR protocols [11]. HPV156 was initially detected in a sun-exposed healthy skin specimen [6], while HPV172 was found in an oral rinse sample of a healthy individual [55]. Based on these data, it seems that at least some of the closest relatives of HPV179 and HPV184 share both cutaneous and mucosal tissue tropism, with some HPV types being causally related to the development of benign lesions.

In order to assess the biological and potential clinical importance of the two novel HPV types, a representative collection of HPV-associated benign and malignant neoplasms, hair follicles and anal and oral mucosa specimens were tested using HPV179 and HPV184 type-specific quantitative RT-PCR assays. The sample collection was designed to represent known sites of HPV infection (mucosal, cutaneous or mucocutaneous) and important HPV-related benign or malignant lesions (Tables 3 and 4) [38]. The sample panel thus included the most important HPV-associated malignant neoplasms of mucosal (cervical and oral/oropharyngeal SCC) and cutaneous (SCC and BCC of skin) origin, and the most important HPV-associated benign neoplasms of mucosal (genital warts, laryngeal and conjunctival papillomas) and cutaneous (common warts) origin. Hair follicles and swabs of the oral cavity were additionally tested to determine the prevalence of HPV179 and HPV184 in clinically healthy skin and oral mucosa, which are both known as reservoirs of various *Gamma-*PV types in immunocompetent individuals [14, 15, 53]. Swabs of the anal canal, some from patients with HPV6/HPV11 positive anal warts, were also tested to see whether HPV179 and HPV184 are able to infect mucocutaneous epithelia. In addition, in all HPV179 and/or HPV184 positive samples, the HPV179 and/or HPV184 viral load per human cell was calculated in order to estimate their role in the development of various neoplasms. Namely, it has been shown previously that the viral load of *Alpha*-PV types, such as HPV3, HPV27, and HPV57, commonly involved in the development of a cutaneous wart, is generally high, ranging from 50 to more than 3x10^5^ viruses per cell, and is independent of the immune status of the host [19]. However, to the best of our knowledge, no viral load has been determined so far for *Gamma-*PV types potentially involved in the development of skin neoplasms. All water blanks used to control for PCR contaminations were negative for HPV DNA with all PCR tests.

**Table 3:** Clinical samples tested for the presence of HPV179 and characteristics of HPV179 positive samples.

Tissue type	Histology (sample type)	No. of samples tested	No. of HPV179-positives	Prevalence of HPV179 (%)	Sample no.	HPV179 viral load (viral copies/10^4^ cells)	Presence of other HPVs
**Mucosal**	Oral cavity (swabs)	50	0	0	/	/	/
	Oral and oropharyngeal SCC (FFPE)	50	0	0	/	/	/
	Laryngeal papilloma (fresh tissue)	31	0	0	/	/	/
	Conjuctival papilloma (FFPE)	31	0	0	/	/	/
	Cervical cancer	31	0	0	/	/	/
**Cutaneous**	Common warts (FFPE, fresh tissue)	94	4	4.2	S1	2.0	1, 2, 5, 36
					S2	1.2	2, 4, 5, 9,12, 17, 24, 93
					S3	1.2	2, 12, 24, 76, 93
					S4	11	4
	SCC (FFPE)	50	1	2.0	S5	1,400	9, 21, 24, 36, 151
	BCC (FFPE)	51	1	2.0	S6	32	9, 17, 23, 24, 38, 92, 93
	Eyebrows (hair follicles)	100	1	1.0	S7	91	22, 38
	Condyloma acuminatum (fresh tissue)	31	0	0	/	/	/
**Mucocutaneous**	Anal canal (swabs)	50	0	0	/	/	/
**Total**		569	7	1.23			

Abbreviations/legend: SCC—squamous cell carcinoma. FFPE—formalin-fixed paraffin-embedded tissues.

**Table 4:** Clinical samples tested for the presence of HPV184 and characteristics of HPV184 positive samples.

Tissue type	Histology (sample type)	No. of samples tested	No. of HPV184-positives	Prevalence of HPV184 (%)	Sample no.	HPV184 viral load (viral copies/10^4^ cells)	Presence of other HPVs
**Mucosal**	Oral cavity (swabs)	50	0	0	/	/	/
	Oral and oropharyngeal SCC (FFPE)	50	1	2.0	S8	88	36
	Laryngeal papilloma (fresh tissue)	31	0	0	/	/	/
	Conjuctival papilloma (FFPE)	31	0	0	/	/	/
	Cervical cancer	31	0	0	/	/	/
**Cutaneous**	Common warts (FFPE, fresh tissue)	94	0	0	/	/	/
	SCC (FFPE)	50	0	0	/	/	/
	BCC (FFPE)	51	0	0	/	/	/
	Eyebrows (hair follicles)	100	3	3.0	S9	ND	15
					S10	47	12, 23, 49, 80, 93
					S11	85	8, 12, 23, 24, 36, 38
	Condyloma acuminatum (fresh tissue)	31	0	0	/	/	/
**Mucocutaneous**	Anal canal (swabs)	50	0	0	/	/	/
**Total**		569	4	0.70			

Abbreviations/legend: SCC—squamous cell carcinoma. FFPE—formalin-fixed paraffin-embedded tissues. ND—not done.

As shown in Table 1, in the original HPV179 positive facial wart tissue specimen (left cheek), the HPV179 viral load was estimated to be 2,463 genome copies per single cell. This particular specimen additionally contained multiple *Beta-*PV types, including HPV8, -9, -12, -14, -15, -20, -23, -24, -49, -75, -80, -93, -174 and a putative subtype of HPV150 [38]. Using a HPV184 type specific RT-PCR assay, HPV184 was also detected in low quantities (0.40 viral copies/cell), probably representing a cross contamination from the original HPV184 positive wart located on the patient’s right cheek. In the original HPV184 positive facial wart tissue specimen, the HPV184 viral load was similarly estimated to several thousand genome copies (3,200) per single cell. This particular specimen additionally contained several *Beta-*PV types: HPV8, -9, -23, -49, -174, and a putative subtype of HPV150. Using HPV179 type specific RT-PCR assay, HPV179 was also detected in low quantities (0.30 viral copies/cell), probably representing a cross contamination from the original HPV179 positive wart located on the patient’s left cheek. The relatively high viral loads of HPV179 and HPV184 and the absence of common wart-associated HPV types in patients’ facial warts strongly suggest a possible active role of the novel HPV types in the development of these particular lesions.

During a follow-up examination of the patient, eight years after transplantation and four years after the initial removal of facial common warts, several skin specimens were collected, including a newly formed common wart on a neck, and tested for the presence of HPV179 and HPV184 using type-specific RT-PCR assays. As shown in Table 1, HPV179 and HPV184 were detected at the original sites of the resolved facial warts and all other distantly related anatomical sites, indicating that both novel HPV types can establish long-term, persistent infection of the skin for at least four years. It has been previously shown that HPV infections are more persistent in immunosuppressed patients, in comparison to immunocompetent patients, and that the transmission of HPV types to other body locations, due to autoinoculation, is likely to happen [56, 57]. Interestingly, both novel HPV types were also detected in a neck common wart, histologically classified as *verruca vulgaris*. However, viral loads of HPV179 and HPV184 in this particular wart tissue specimen were lower than in the initial facial wart tissue specimens (0.01 vs. 2,463 viral copies/cell for HPV179, and 0.20 vs. 3,200 viral copies/cell for HPV184), suggesting that neither HPV179 nor HPV184 was the causative agent of the newly formed common wart. As additionally shown in Table 1, no other cutaneous wart-associated HPV types were detected in the common wart from the neck, suggesting a possible etiological role of other, still undiscovered HPVs.

Using a HPV179 type specific RT-PCR assay, HPV179 was further detected in low-copy numbers (1–1,400 viral genomes/10^4^ cells) in various clinical samples, including 4/94 (4.2%) samples of common warts, 1/50 (2.0%) samples of skin SCC, 1/51 (2.0%) samples of skin BCC and 1/100 (1.0%) samples of eyebrow hair follicles (Table 3). All HPV179 positive SCC and BCC of the skin and eyebrows additionally contained several *Beta-*PV types. Similarly, all HPV179 positive warts contained several *Beta-*PV types, and additionally HPV1, HPV2 and/or HPV4, which we strongly believe were the true etiological agents of these specific neoplasms (Table 3). Interestingly, none of the 243 mucosal or mucocutaneous samples were positive for HPV179, suggesting a strictly cutaneous tropism of this HPV type. However, more mucosal samples should be screened for HPV179 to draw final conclusions. Although the role of *Beta*-PV in the development of BCC and SCC of the skin is not yet fully understood, both experimental and epidemiological evidence suggest a carcinogenic role of *Beta*-PV types in these types of skin cancer [58]. In contrast, there is currently no published evidence that *Gamma-*PV types play an important role in the development of skin cancer. However, the results of present and some previous studies [2, 18–22] have undoubtedly shown that *Gamma-*PVs can be etiologically linked to sporadic cases of common warts, especially in immunosuppressed patients. In contrast, it seems that *Beta*-PV types are not important etiological agents of cutaneous warts but rather their superficial or tumor surrounding tissue contaminants. As shown here and in the study of Kohler et al., in cutaneous warts, *Beta*-PV types are typically co-present with cutaneous wart-associated HPV types, with viral loads being extremely low, usually less than 5,700 viral genomes per 10^4^ cells [19].

Using an HPV184 type specific RT-PCR assay, HPV184 was further detected in low-copy numbers (47–88 viral genomes/10^4^ cells) in only 1/50 (2.0%) samples of oral/oropharyngeal SCC and 3/100 (1.0%) samples of eyebrow hair follicles, indicating the mucosal and cutaneous tropism of this HPV type (Table 4). As with HPV179, all HPV184 positive samples additionally contained one to six different *Beta-*PV types, thus further confirming that skin epithelium, especially hair follicles, can host HPV types from different PV-genera [25, 43, 59]. According to recent suggestions, persistent infection with high-risk *Alpha*-PV types (especially with HPV16) is associated with a subgroup of cancers of the oral cavity and oropharynx [60]. However, recent reports indicate that a wide spectrum of HPV types from the *Beta*-PV and *Gamma*-PV genera can be detected in the oral cavity and/or larynx of healthy individuals [15, 20, 27]. In addition, we have previously shown that *Beta*-PV types can also be found in the verrucous carcinoma of the oral cavity and larynx, thus raising the question of whether and how mechanistically *Beta*-PVs and *Gamma*-PVs participate in the development of mucosal malignant tumors [27]. Interestingly, the calculated viral load of HPV184 in the oral SCC in this study was approximately 9 viral genomes per 1,000 tumor cells, typical of what is usually seen for *Beta*-PV types present in SCC of the skin [19].

We are aware that DNA in FFPE tissue specimens is often degraded and that the majority of *Gamma*-PV infections are probably latent, characterized by low-copy numbers. Therefore, the HPV179 and HPV184 prevalences and viral loads in clinical samples represented solely by the FFPE tissues specimens might be underestimated.

## Conclusions

Two novel *Gammapapillomaviruses*, HPV179 and HPV184, were identified in common warts that developed on the face of a renal-transplant recipient. Both novel viruses exhibited genome organization and characteristics typical of *Gamma-*PVs. Phylogenetically, HPV179 was classified as a novel member of species *Gamma*-15, additionally containing HPV135 and HPV146, while HPV184 was classified as a single member of a novel species *Gamma*-*2*5. HPV179 and HPV184 are relatively rare HPV types, with cutaneous and mucosal/cutaneous tissue tropism, respectively, etiologically linked with sporadic cases of common warts, probably more often in immunosuppressed patients. Both novel HPV types established a persistent infection of the skin for more than four years.
